# A Highly Sensitive Quantitative Real-Time PCR Assay for Determination of Mutant *JAK2* Exon 12 Allele Burden

**DOI:** 10.1371/journal.pone.0033100

**Published:** 2012-03-05

**Authors:** Lasse Kjær, Maj Westman, Caroline Hasselbalch Riley, Estrid Høgdall, Ole Weis Bjerrum, Hans Hasselbalch

**Affiliations:** 1 Department of Hematology, Herlev Hospital, Herlev, Denmark; 2 Department of Clinical Genetics, Rigshospitalet, Copenhagen, Denmark; 3 Department of Pathology, Herlev Hospital, Herlev, Denmark; 4 Department of Hematology, Rigshospitalet, Copenhagen, Denmark; 5 Department of Hematology, Roskilde Hospital, Copenhagen, Denmark; Lund University Hospital, Sweden

## Abstract

Mutations in the *Janus kinase 2* (*JAK2*) gene have become an important identifier for the Philadelphia-chromosome negative chronic myeloproliferative neoplasms. In contrast to the *JAK2*V617F mutation, the large number of *JAK2* exon 12 mutations has challenged the development of quantitative assays. We present a highly sensitive real-time quantitative PCR assay for determination of the mutant allele burden of *JAK2* exon 12 mutations. In combination with high resolution melting analysis and sequencing the assay identified six patients carrying previously described *JAK2* exon 12 mutations and one novel mutation. Two patients were homozygous with a high mutant allele burden, whereas one of the heterozygous patients had a very low mutant allele burden. The allele burden in the peripheral blood resembled that of the bone marrow, except for the patient with low allele burden. Myeloid and lymphoid cell populations were isolated by cell sorting and quantitative PCR revealed similar mutant allele burdens in CD16+ granulocytes and peripheral blood. The mutations were also detected in B-lymphocytes in half of the patients at a low allele burden. In conclusion, our highly sensitive assay provides an important tool for quantitative monitoring of the mutant allele burden and accordingly also for determining the impact of treatment with interferon-α-2, shown to induce molecular remission in *JAK2*V617F-positive patients, which may be a future treatment option for *JAK2* exon 12-positive patients as well.

## Introduction

Somatic mutations in the *Janus kinase 2* (*JAK2*) gene contribute significantly to the pathogenesis of Philadelphia chromosome –negative chronic myeloproliferative neoplasms (MPN). In particular, polycythemia vera (PV) is in most cases associated with the *JAK2*V617F mutation. As a consequence, constitutive phosphorylation of JAK2 results in activation of downstream signalling pathways, leading to cytokine-independent growth of haematopoietic progenitors and increased production of cells of the myeloid lineage [1]. The identification of JAK2 as a therapeutic target in the treatment of MPNs has been a major contribution towards the understanding and clinical management of these diseases [2]–[6]. Several studies have shown that small molecule JAK2-inhibitors affect growth and viability of the neoplastic cells but also possess potent anti-inflammatory effects and display remarkable resolution of hypermetabolic symptoms in concert with reduction of splenomegaly in patients with myelofibrosis (MF) [7]–[13]. Although significant reduction of the mutant allele burden has been reported in a subgroup of patients [13] the JAK2-inhibitors in general do not influence the *JAK2*V617F allele burden [14], [15]. The presence of the *JAK2*V617F mutation has been established as a risk factor for thrombosis [16], but the relationship between mutant allele burden and the risk of thrombosis remains controversial [17]–[19]. However, the observations that the *JAK2*V617F mutation per se induces increasing genetic instability [20], in addition to the association between the *JAK2*V617F mutant allele burden and both disease phenotype and MF-free survival, support the contention of early intervention with agents having the potential to reduce the malignant clone [18]. In this context, interferon (IFN)-α-2 (2a and 2b), has been shown to induce major - and in a subset of patients also - complete molecular remissions in *JAK2*V617F-positive patients with essential thrombocytosis (ET) and PV [21]. Patients with *JAK2* exon 12 positive PV appear to progress along a clinical course similar to *JAK2*V617F -positive PV with regard to risk factors, including the development of thrombosis and post-PV MF [22]. This observation suggests IFN-α-2 to be a rational therapeutic approach aiming at inducing minimal residual disease with low-burden JAK2 exon 12 as well. Monitoring the impact of treatment upon the neoplastic clone requires a robust method to determine the mutant allele burden with high sensitivity in order to be able to assess deep molecular remissions. For the purpose of monitoring the mutant allele burden of the *JAK2*V617F mutation sensitive, quantitative PCR (qPCR) assays have been developed [23], [24]. As opposed to the single nucleotide substitution of *JAK2*V617F, at least 37 different *JAK2* exon 12 mutations have been described, residing in a region involving amino acids V536-F547 [25]. For identification of *JAK2* exon 12 mutations, high resolution melting (HRM) analysis techniques have emerged as superior to both common allele-specific PCR assays and Sanger sequencing in sensitivity and convenience for screening of clinical samples [26]–[28]. Certain assays have demonstrated high sensitivity for selected *JAK2* exon 12 mutations [26], [29], but the large amount and variability of mutations have complicated the development of quantitative assays necessary for the evaluation of remission -inducing agents. In the present study we have developed a highly sensitive quantitative real-time qPCR technique for the most frequently occurring *JAK2* exon 12 mutations and used this assay to investigate the proportion of mutated cells in different peripheral blood (PB) cell lineages of *JAK2* exon 12 positive patients. In addition, a novel *JAK2* exon 12 mutation is reported.

## Results

### Identification of the JAK2 exon 12 mutations

**Figure 1:**
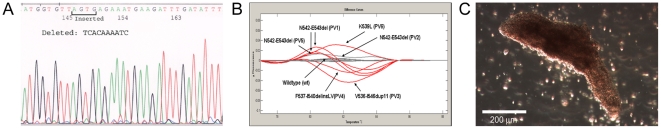
Identification of *JAK2* exon 12 mutations. A: Sequence of F537-I540delinsLV mutation of PV4 revealing a 10 base-pair deletion and a four base-pair insertion. B: Difference plot of high resolution melting (HRM) analysis detecting N542-E543del, F537-I540delinsLV and K539L *JAK2* exon 12 mutations in PV1, PV3, PV4, PV5 and PV6. C: Phase-contrast microphotograph showing Epo-independent growth of endogenous erythroid colonies (EEC) at day 14 of culture. Scalebar: 200 µm. HRM, high resolution melting.

In cohort 1, four patients (6.7%, n = 60) were found *JAK2* exon 12 positive (PV1-PV4) (Table S1). Two patients were identified with the N542-E543del mutation by qPCR screening, one patient with a V536-I546dup11 mutation and one patient with a novel mutation involving a 10 base-pair deletion and a four base-pair insertion; F537-I540delinsLV were identified by Sanger sequencing (Figure 1A). In cohort 2, HRM analysis identified two patients with *JAK2* exon 12 mutations (1.96%, n = 102): one patient with a N542-E543del (PV5) and one patient with a K539L mutation (PV6) (Figure 1B). The mutations identified by qPCR and Sanger sequencing were validated by HRM analysis and the mutations identified in patients of cohort 2 were validated by qPCR and Sanger sequencing. Neither Sanger sequencing nor HRM could confirm the N542-E543del mutation of PV2 identified by qPCR (Figure 1B). Consistent with these observations Epo-independent Erythroid Colony (EEC) -growth was detected in cultures of mononuclear cells from all patients except PV2 (Figure 1C and Table S1).

### Clinical characteristics of patients carrying JAK2 exon 12 mutations

The patients included three male and three female patients with median age of 64 years (range 20–88). PV1, PV2 and PV4 had enlarged spleens at the time of diagnosis verified on abdominal ultrasound-scan (Table S1). Later on PV3 and PV6 developed enlarged spleens during progression of disease. At the time of diagnosis all patients had elevated hematocrit (median 0.6; range 0.56–0.81) and normal platelet counts and one patient had persistent leukocytosis (PV4). Bone marrow (BM) biopsies were hypercellular with characteristics consistent with MPN in 5 patients. BM evaluation was not performed in PV6. None had a history of thrombosis at the time of diagnosis, but occurred at a later time in PV2 (not shown). All patients were initially treated with phlebotomy and aspirin in addition; both PV2 and PV4 needed supplementary cytoreductive therapy with hydroxyurea due to thrombosis and leukocytosis, respectively (Table S1).

### Quantitative determination of mutant JAK2 exon 12 allele burden

**Figure 2:**
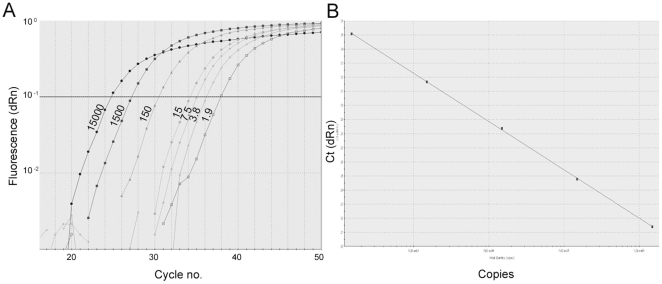
Amplification plots of dilution series and standard curve of qPCR assay. A: Representative amplification plots of plasmid containing *JAK2* exon 12 mutation diluted into wildtype genomic DNA detected by the mutation specific assay. The ten fold dilution series starting at 15,000 copies is continued as two-fold dilutions after 15 copies down to 1.9 copies as indicated. B: Representative standard curve for the mutation and wildtype assays producing correlation coefficients >0.990. The average slope of the standard curves was −3.4.

The correlation coefficients of standard curves were all above 0.990 and an average slope of −3.4 was determined from several standard curve experiments (Figure 2A). The Y-intercept corresponding to one target gene copy was determined for each primer set by series of two-fold dilutions of both genomic DNA (gDNA) and plasmids. The mutant copy number (*JAK2_mut_*) was then determined by the equation 10∧(Y-intercept−Ct*_JAK2mut_*/−slope*_JAK2mut_*) and the wildtype (wt) copy number (*JAK2_wt_*) by the equation 10∧(Y-intercept−Ct*_JAK2wt_*/−slope*_JAK2wt_*). The percentage mutant allele burden was then calculated as (Copy number (*JAK2_mut_*)/[Copy number (*JAK2_mut_*)+Copy number (*JAK2_wt_*)])×100.

Cross-reaction with the wt sequence of the mutant specific assay was determined by amplification of gDNA pooled from 42 healthy donors. Unspecific amplification occasionally occurred for the V536-I540dup11 primers but always with a difference in Ct values of at least 18 Cts corresponding to a sensitivity of at least 1/(1.93^18^) = 0.0007% (not shown). For significant detection of the mutation a 10 fold higher cut-off was set to a maximal sensitivity for the V536-I540dup11 mutation of 0.007%. The sensitivity of the individual samples was calculated from the equation (1/[Copy number (*JAK2_mut_*)+Copy number (*JAK2_wt_*)])×100. (Tables S2, S3, S4, S5). Plasmids containing the mutated sequence were diluted into wt gDNA and least 1.9 mutant copies could be detected in a total of 15.000 *JAK2* copies corresponding to 0.01% mutated alleles (Figure 2B). The sensitivities for the samples were below 0.01% except for a few samples with limited amount of DNA (Tables S2, S3, S4, S5).

**Figure 3:**
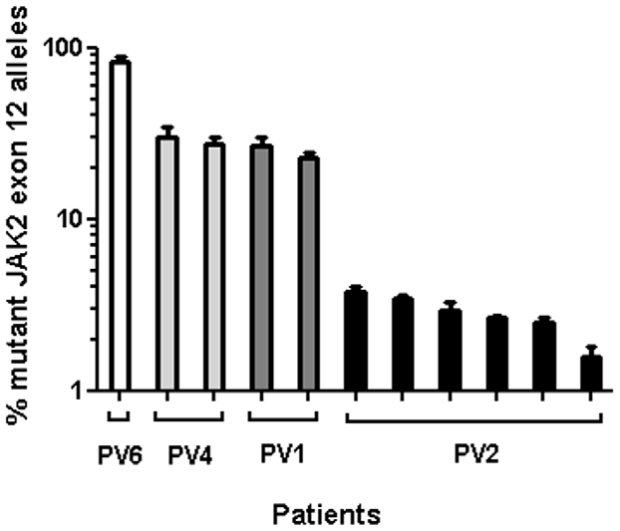
Reproducibility of mutant allele burden determination by the qPCR assays. Histogram plots showing reproducibility of percentage mutant allele burden in six separate qPCR runs. The mutant allele burden was determined for 11 different DNA samples from four different patients as indicated. The *JAK2* exon 12 mutations include K539L (PV6), V536-I546dup11 (PV4), and N542-E543del (PV1 and PV2) in high, intermediate and low levels of mutant allele burden. The data is presented as percentage mean values ± standard deviation (SD).

To ensure that the assays demonstrated reproducibility suitable for clinical applications eleven patient samples including high, intermediate, and low mutant allele burdens were analysed from six separate qPCR runs. The assays were highly reproducible with no more than a 1.5 fold difference recorded within a dataset (Figure 3 and Table S6).

### Mutant allele burden in patient samples

The patients with K539L and the novel F537-I540delinsLV mutations (PV4 and PV6, respectively) had a remarkably high homozygous allele burden of 89% and 99% respectively (Supplementary Tables S1 and S5). The heterozygous patients PV1, PV3 and PV5 had low intermediate to intermediate levels of mutant allele burdens in the range of 17–33%, whereas PV2 had a very low mutant allele burden ranging from of 1.5–3.4% (Tables S1, S2, S3, S4, S5).

**Figure 4:**
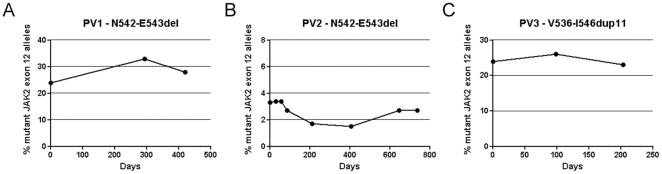
Time-course of *JAK2* exon 12 allele burden development. A and B: Graphs describing the development of the N542-E543del mutant allele burdens of PV1 and PV2 during a 420 and 735 day period respectively. C: Plot of V536-I546dup11 mutant allele burden of PV3 during a 203 day period.

To investigate the development of the mutated clones, variations in mutant allele burdens were investigated for PV1, PV2, and PV3 for 420, 735, and 203 days, respectively. The mutant allele burdens were stable over time for the intermediate and low level mutant allele burdens investigated (Figure 4 and Tables S2, S3, and S4).

**Figure 5:**
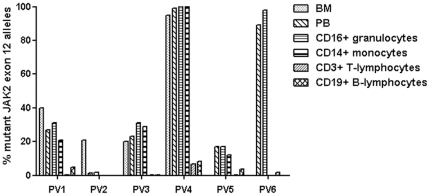
Mutant *JAK2* exon 12 allele burden in bone marrow and peripheral blood cell lineages. Histogram plot displaying the *JAK2* exon 12 mutation burden in bone marrow, peripheral blood, CD16^+^ granulocytes, CD14^+^ monocytes, CD3^+^ T-lymphocytes, and CD19^+^ B-lymphocytes in patients PV1-PV6. PV2 had very low level of *JAK2* exon 12 mutant allele burden except for the bone marrow sample. PV1-PV3 and PV5 appeared to be heterozygous, whereas PV4 and PV6 were homozygous. Note that DNA from bone marrow samples could not be obtained from patients PV5 and PV6. BM, bone marrow and PB, peripheral blood.

Analysis of DNA extracted from paraffin embedded BM samples resulted in sensitivities in the range of 0.007–0.016% (Table S5). This was presumably the result of lower concentrations of DNA and fragmentation following decalcifying acid treatment of the samples. Consequently, no usable DNA could be obtained from BM of patients PV5 and PV6. The mutant allele burden in BM was slightly higher than in the PB in PV1 (40% in BM compared to 24–33% in PB), but similar for PV3 (17% in BM compared to 19–22% in PB) and PV4 (95% in BM compared to 99% in PB), whereas the mutant allele burden in PV2 was significantly higher in the BM sample (21% in BM compared to 1.5% in PB) (Figure 5 and Table S5).

### Cell sorting

The allele burden of the *JAK2* exon 12 mutation was analysed by qPCR for the granulocyte, monocyte, B-lymphocyte and T-lymphocyte cell populations isolated by fluorescence activated cell sorting (FACS) (Figure S1). Limitations in cell number in the sorted fractions resulted in sensitivities up to 2.1% (Table S5). However, all were below the sorting purities of the isolated cell fractions, defining lower detection limits for CD16 positive granulocytes and CD19 positive B-lymphocytes to 2% mutated alleles, CD3 positive T-lymphocytes to 7% mutated alleles and CD14 positive monocytes to 8% mutated alleles. The mutant allele burden of the granulocyte fraction closely resembled that of PB (Figure 5). The allele burden in the monocyte compartment also resembled that seen in PB, except for PV2 where no mutation could be detected, and PV5 where no mutation was detected as a result of very low levels of DNA for this fraction (Table S3). *JAK2* exon 12 mutations were detected in B-lymphocytes in half of the patients with allele burdens above the detection limit, but not in T-lymphocytes as the mutant allele burden of 6.6% in PV4 could stem from sorting impurities (Figure 5).

## Discussion

In the present study, a highly sensitive qPCR assay was developed for assessing the allele burden of the identified exon 12 mutations in PB and cell populations isolated from PB. The method improves the current sensitivity for detection of exon 12 mutations and enables monitoring of the mutational status even for very low mutant allele burdens.

Two different strategies were established to screen for *JAK2* exon 12 mutations in patients with MPN: qPCR or HRM combined with Sanger sequencing. Sanger sequencing is a reliable technique when investigating a wide variety of mutations but has a detection limit of 10–20% mutated alleles. Various HRM assays have been developed with sensitivities depending on the assay and mutation (1–20%) [26]–[28], [30]. As it has been suggested that there are no clinical differences among the *JAK2* exon 12 mutation variants [22], HRM analysis may at present be the best techniques for investigation of routine samples, but is not sufficiently sensitive as a pre-qPCR screen. A recent technique combining clamped PCR with fragment analysis revealed the identity of the mutated sequence and had a sensitivity of 0.1%, but this was only investigated for the N542-E543del mutation [29]. The qPCR assay of the present study is at least ten fold more sensitive. Using a less sensitive technique to screen samples for *JAK2* exon 12 mutations, would abrogate the superior sensitivity of the developed qPCR technique in identifying patients with very low allele burden. Accordingly, using qPCR we screened for eight of the most common exon 12 mutations representing at least 62% of all reported *JAK2* exon 12 mutations [25] and 85% of *JAK2* exon 12 mutations of a recent European multicenter study [22]. The necessity for subsequent Sanger sequencing of the samples to identify mutations not included in the initial screen was highlighted by identification of the two patients carrying the V536-I546dup11 and the F537-I540delinsLV mutations. To the authors knowledge the F537-I540delinsLV mutation has not been described previously.

It could be argued that using a HRM assay is more convenient for large scale routine screening, but using only this technique the N542-E543del mutation of PV2 in the present study would have eluded detection. Furthermore determining the exact identity of a mutation can be very challenging or impossible when using HRM on samples with a mutant allele burden below the detection limit of Sanger sequencing.

The risk of false positives is an important issue, especially when using very sensitive techniques and confirmation of results obtained by a technique with unrivalled sensitivity is inherently difficult. The N542-E543del mutation of PV2 was the only mutation detected by qPCR which could not reliably be confirmed as a true positive by the other techniques. However, the eight separate DNA samples of PV2 were isolated over a period of 735 days and were positive for the mutation in a total of 37 triplicates performed in 11 separate plates, run on different days. This strongly suggests that PV2 is indeed a true positive.

The mutant allele ratios of PV1, PV2 and PV3 were stable over time, resembling observations in *JAK2*V617F patients [31]. The *JAK2* exon 12 allele burden was comparable in PB and BM patient samples, as has been reported earlier for patients carrying the *JAK2*V617F mutation [32].

Interestingly, corresponding to the large discrepancy between mutant allele burden in PB and BM of PV2, Larsen et al., also reported significantly higher mutant allele burden in the BM of a patient with a relatively low *JAK2*V617F mutant allele burden [32]. In the context of using the mutant allele burden as a prognostic factor further studies are needed to clarify if there are patients with inconsistencies between mutant allele burden in the PB and the BM.

The corresponding allele burdens for PB and for the granulocyte fractions suggest that isolation of granulocytes does not decrease the frequency of false negatives, even at very low mutant allele burden, and is thus unnecessary. Instead, “whole blood qPCR” is advocated, which has also been shown to be a highly reliable methodology in assessing the JAK2V617F -allele burden [23], [32]. Exon 12 mutations were detected in B-lymphocytes in half of the patients analysed, although not conclusively in T-lymphocytes, which supports a previous report that these mutations are present in the lymphoid compartment [33]. The reduction of the qPCR sensitivity resulting from the limited amount of cells in the sorted cell fractions was not of any concern, as it did not rise above the sorting purity cut-off for the positive samples. The low allele burden of the B-lymphocytes did not correspond to the allele burden of PB suggesting that the exon 12 mutations do not impose a selective advantage on the differentiation and/or expansion of the lymphoid compared to the myeloid cells. Although leukocyte counts were in the normal range for PV1 and PV5, it is interesting to note that PV4 - the patient with the highest mutant allele burden in B-lymphocytes - had an elevated leukocyte count. Evaluation of the *JAK2* exon 12 mutant allele burden as a prognostic marker, however, requires the investigation of a larger patient material.

Patients with JAK2 exon 12 mutations may proceed along a clinical course very similar to those with the *JAK2*V617- mutation, implying steadily progressive disease with development of MF [22], [34]. In the context that IFN-α-2 is able to induce major and complete molecular remissions in *JAK2*V617F -positive patients it is foreseen that similar results may be achieved in *JAK2* exon 12 positive patients as well. In this setting the present report calls for larger clinical studies evaluating the efficacy of IFN-α-2 in inducing low-burden JAK2 exon 12 in this patient cohort and the impact on disease evolution as well. In conclusion, the sensitive and quantitative technique presented in the current study provides a powerful tool for evaluating the mutant allele burden as a prognostic factor and for monitoring of the mutant allele burden during treatment with IFN-α-2.

## Materials and Methods

### Patients

The study included two cohorts. Cohort 1 consisted of 60 patients with MPN from Herlev hospital, including four patients with idiopathic erythrocytosis (IE) and 56 patients classified according to the World Health Organization (WHO) criteria [1]: 15 ET, 37 PV and 4 primary MF (PMF). Cohort 2 included 102 patients from Rigshospitalet with suspected MPN submitted for routine *JAK2*V617F analysis. All patients were tested negative for the *JAK2*V617F mutation using a sensitive qPCR assay [23]. The procedures used were approved by the Capital Region Committee on Health Research Ethics. The study was carried out according to the declaration of Helsinki.

### DNA analysis and Epo –independent Endogenous Colony growth

DNA from PB was extracted using the Qiaamp DNA mini kit (Qiagen, Hilden, Germany) according to manufacturer's instructions. JAK2 exon 12 mutations in samples from cohort 1 were identified by real-time qPCR screening for the exon 12 mutation variants, in addition to Sanger sequencing as described below. JAK2 exon 12 mutations in cohort 2 samples were identified by HRM analysis followed by subsequent genotyping by sequencing. Samples with a mutation identified either by real-time qPCR, HRM and/or sequencing were investigated for Erythropoietin (Epo) -independent Endogenous Colony (EEC) –growth from PB using MethoCult Agar-LCM according to manufacturer's instructions (Stemcell technologies, Grenoble, France).

### qPCR design

The *JAK2* exon 12 qPCR screening assays were designed for the wt sequence and the mutations: N542-543del, E543-D544del, K539L, F537-K539delinsL, H538-K539delinsL, H538QK539L, R541-E543delinsK, and I540-E543delinsMK. After identification of the mutations by Sanger sequencing (see results) quantitative assays were also designed for the V536-I546dup11 and F537-I540delinsLV mutations. The design included a common forward primer and probe in addition to a wt or mutation specific primer, resulting in a PCR product of approximately 130 bp. To increase the sensitivity a mismatch was introduced at the 3′end of the reverse primers, except for H538-K539delinsL, V536-I546dup11, N542-E543del, and F537-I540delinsLV. For quantitative analysis, a common wt reverse primer was used, except the mutation N542-E543del for which a specific reverse wt primer was designed (Table 1). Standard curves were generated using plasmids containing the exon 12 mutations flanked by 176 bp of the upstream intron and 240 bp of the downstream intron (GeneArt, Regensburg, Germany). The plasmids were transfected into competent E. Coli (Invitrogen, Paisley, United Kingdom) and extracted using Genelute TM HP Endotoxin free plasmid maxiprep kit (Sigma-Aldrich, St. Louis, Missouri). The plasmid sequence was confirmed by sequencing. The qPCR reaction was performed in triplicate on 50 ng gDNA in a volume 25 µl containing 300 ng/µl primers and 100 ng/µl probe. The two-step PCR reaction were initiated by a heat activation step for 10 min at 95°C followed by 50 cycles of 15 s at 95°C and 60 s at 60°C. The qPCR reactions were carried out using the Brilliant II qPCR Master Mix on an MxPro3000 (Agilent, Santa Clara, California). The relevant PCR product was subsequently sequenced for confirmation. Plasmids containing the relevant mutated sequence were used for positive control whereas a pool of gDNA of 42 healthy donors was used as a negative control and to determine unspecific amplification of the wt sequence by the mutant specific assays.

**Table 1:** Primer and probe sequences for *JAK2* exon 12.

Primers	Sequence 5′-3′
*JAK2*exon12-Forw	CAGATAAATCAAACCTTCTAGTCTTC
*JAK2*exon12-wt-Rev	CAAATCTTCATTTCTGATTTTGTtA
*JAK2*exon12-N542-E543del-Rev	TGACTTACAAATATCAAATCTCTGA
*JAK2*exon12-E543-D544del-Rev	CAAATCTTCATTTCTGATTAAGTtA
*JAK2*exon12-K539L-Rev	CAAATCTTCATTTCTGATTAAGTtA
*JAK2*exon12-H538-K539delinsL-Rev	AAATATCAAATCTTCATTTCTGATTAAA
*JAK2*exon12-I540-E543delinsMK-Rev	CAAATATCAAATCTTTCATTTTGTtA
*JAK2*exon12-H538QK539L-Rev	CAAATCTTCATTTCTGATTAATTtA
*JAK2*exon12-R541-E543delinsK-Rev	CAAATATCAAATCTTTGATTTTGTtA
*JAK2*exon12-F537-K539delinsL-Rev	CAAATCTTCATTTCTGATTAACACtA
**Primers only for quantitative determination**	
*JAK2*exon12-wt (N542-E543del)-Rev	AATGACTTACAAATATCAAATCTTCA
*JAK2*exon12-V536-I546dup11-Rev	TTTGTGAAACACTATCAAATCTTC
*JAK2*exon12-F537-I540delinsLV-Rev	AAATATCAAATCTTCATTTCTCACTA
**Probe**	
*JAK2*exon12 probe	Fam-CCAACCTCACCAACATTACAGAGGCC-Tamra

Primers used for qPCR screening and quantitative determination consisting of a common forward primer and probe in addition to a mutation specific reverse primer. The primers listed for quantitative determination were only used exclusively for determination of mutant allele burden. Lowercase letters in sequences indicate intended mismatches.

### High resolution melting (HRM) design

A 164 amplicon was generated using M13-tagged primers in *JAK2* exon 12 (5′-CCAACCTCACCAACATTACAGA-3′) and intron 12 (5′- CCAATGTCACATGAATGTAAATCAA-3′). PCR-reactions were performed in a 15 µl volume and included 1× Qiagen PCR Buffer (Qiagen), 1× LC-Green Plus (Idaho Technologies, Salt Lake City, Utah), 200 µM dNTPs, 0.2 µM of each primer, 0.025 U HotStarTaq DNA Polymerase (Qiagen) and approximately 40 ng DNA. Amplification was achieved by touchdown PCR performed on a GeneAmp 2700 (Applied Biosystems, Foster City, California). Heteroduplexes were generated by raising the temperature of the PCR products to 95°C for 30 seconds and allowing the PCR products to slowly cool to room temperature. Samples were subsequently transferred to the Light Scanner instrument (Idaho Technologies) for HRM analysis. To increase the ability to detect homozygous variants, sample mixing was used to generate artificial heteroduplexes as described by Vossen et al., [35]. After sample mixing the samples were again analysed on the Light Scanner instrument.

### Sanger sequencing of cohort 1 and cohort 2 samples

Cohort 1 samples were sequenced using the Big Dye Terminator v1.1 Cycle Sequencing kit (Applied Biosystems) on an ABI 3100 Genetic analyzer (Applied Biosystems).

Samples in cohort 2 where HRM analysis indicated possible sequence variation were directly purified with Exo-SAP (Fermentas, St. Leon-Rot, Germany) and then sequenced.

### Extraction of DNA from paraffin-embedded tissues

DNA was extracted from 20 10 µm thick paraffin bone marrow sections that where initially deparaffinised by xylene followed by 2×96% EtOH. Tissues were digested by overnight treatment with proteinase K in ATL buffer (Qiagen) and DNA was extracted using the QiaAmp DNA mini kit (Qiagen) according to manufacturer's instructions.

### Cell sorting

Red blood cells of PB were lysed using Ortho-Mune Lysis Solution (Herlev Hospital pharmacy, Herlev, Denmark). For isolation of granulocytes and T-lymphocytes, the cells were labelled with monoclonal CD16-fluorescein isothiocyanate (FITC) (DAKO, Glostrup, Denmark) and CD3- phycoerythrin (PE) (BD Biosciences, San Jose, California). Prior to monocyte and B-lymphocyte sorting, peripheral blood mononuclear cells (PBMC)s were prepared using Leucosep according to manufacturer's instructions (Greiner bio-one, Frichenhausen, Germany) and then labelled with monoclonal CD19-FITC and CD14-PE (BD Biosciences). The cells were sorted on a FACSAria (BD biosciences) in purity mode. Analysis of sorted cells revealed purities of 98% for CD16 positive granulocytes, 93% for CD3 positive T-lymphocytes, 98% for the CD19 positive B-lymphocytes, and 92% for CD14 positive monocytes.

## Supporting Information

Figure S1 **Scatter plots of gating strategy for cell sorting.** Scatter plots for gating strategy employed for isolation of A: CD16-FITC, B: CD3-PE, C: CD19-FITC, and D: CD14-PE performed as double labelled sorting with gates for the isolation of CD16^+^ granulocytes and CD3^+^ T-lymphocytes from peripheral blood (blood) and in CD14^+^ monocytes and CD19^+^ B-lymphocytes isolated from peripheral blood mononuclear cells (PBMC). Back gating for separation of labelled cells and single cell isolation in forward- and side scatter plots is illustrated with colours (CD16^+^/CD3^+^ singlets: blue/pink, CD19^+^/CD14^+^ singlets: blue/pink. Cells were gated for cell type on FSC/SSC plot prior to flourophore gating. Singlets were obtained by gating in FSC-H/FSC-A and subsequently SSC-H/SSC-A. PBMC, peripheral blood mononuclear cells.
(TIF)

Table S1 **Clinical features of PV patients with** ***JAK2*** **exon 12 mutations.** Clinical data at time of diagnosis as indicated. Cell counts are in ×10^9^/L. F, female; M, Male; Diagn., diagnosis; PV, polycythemia vera; Years, years from diagnosis; %mut, *JAK2* exon 12 mutant allele burden at time of cell sorting; Hct, hematocrit; Hb, haemoglobin; Wbc, white blood cell count; Trc, platelet count; Epo, erythropoietin; Norm., normal; Spleen, spenomegaly; Thromb, previous thromboses; BM, bone marrow; EEC, Epo-independent Endogenous Colony -growth; Seq, sequencing; Treat, treatment; Hu, hydroxyurea; V, venesection; A, Aspirin. * PV3 and PV6 later developed splenomegaly and PV6 had an incidence of transient cerebral ischemic attack.
(PPT)

Table S2 **Development of N542-E543del mutant allele burden in PV1.** Data for PV1 displaying the percentage mutant allele burden of *JAK2* exon 12 mutations during a time period of 420 days, depicted in Figure 3. Mut (%), percentage mutant allele burden and Sens (%), percentage sensitivity.
(XLS)

Table S3 **Development of N542-E543del mutant allele burden in PV2.** Data for PV2 displaying the percentage mutant allele burden of the *JAK2* exon 12 mutation during a time period of 735 days, depicted in Figure 4. Mut (%), percentage mutant allele burden and Sens (%), percentage sensitivity.
(XLS)

Table S4 **Development of V536-I546dup11 mutant allele burden in PV3.** Data for PV3 displaying the percentage mutant allele burden of *JAK2* exon 12 mutations during a time period of 203 days, depicted in Figure 4. Mut (%), percentage mutant allele burden and Sens (%), percentage sensitivity.
(XLS)

Table S5 **qPCR data of bone marrow, peripheral blood and sorted cell fractions.** Data for patients PV1-PV6 presented in histogram plots shown in Figure 5. showing percentage mutated *JAK2* exon 12 alleles of BM, PB, and sorted cell fractions (CD16^+^ granulocytes, CD14^+^ monocytes, CD3^+^ T-lymphocytes, and CD19^+^ B-lymphocytes). Furthermore the sensitivities are shown calculated as described. BM, bone marrow; PB, peripheral blood; neg., negative; n/a, not available.
(XLS)

Table S6 **Reproducibility analysis of qPCR assay.** Values from reproducibility assay histogram plots presented in Figure 3. The column row indicates the histogram columns of Figure 3. The identity of the mutations for the individual patients in addition to coefficient of variation and mean values ± standard deviations (SD) are shown.
(XLS)
