# Supplementary figures and images for: A Highly Sensitive Quantitative Real-Time PCR Assay for Determination of Mutant JAK2 Exon 12 Allele Burden
Source: PLoS One. 2012 Mar 5;7(3):e33100. doi: 10.1371/journal.pone.0033100 (PMC3293922; doi:10.1371/journal.pone.0033100)

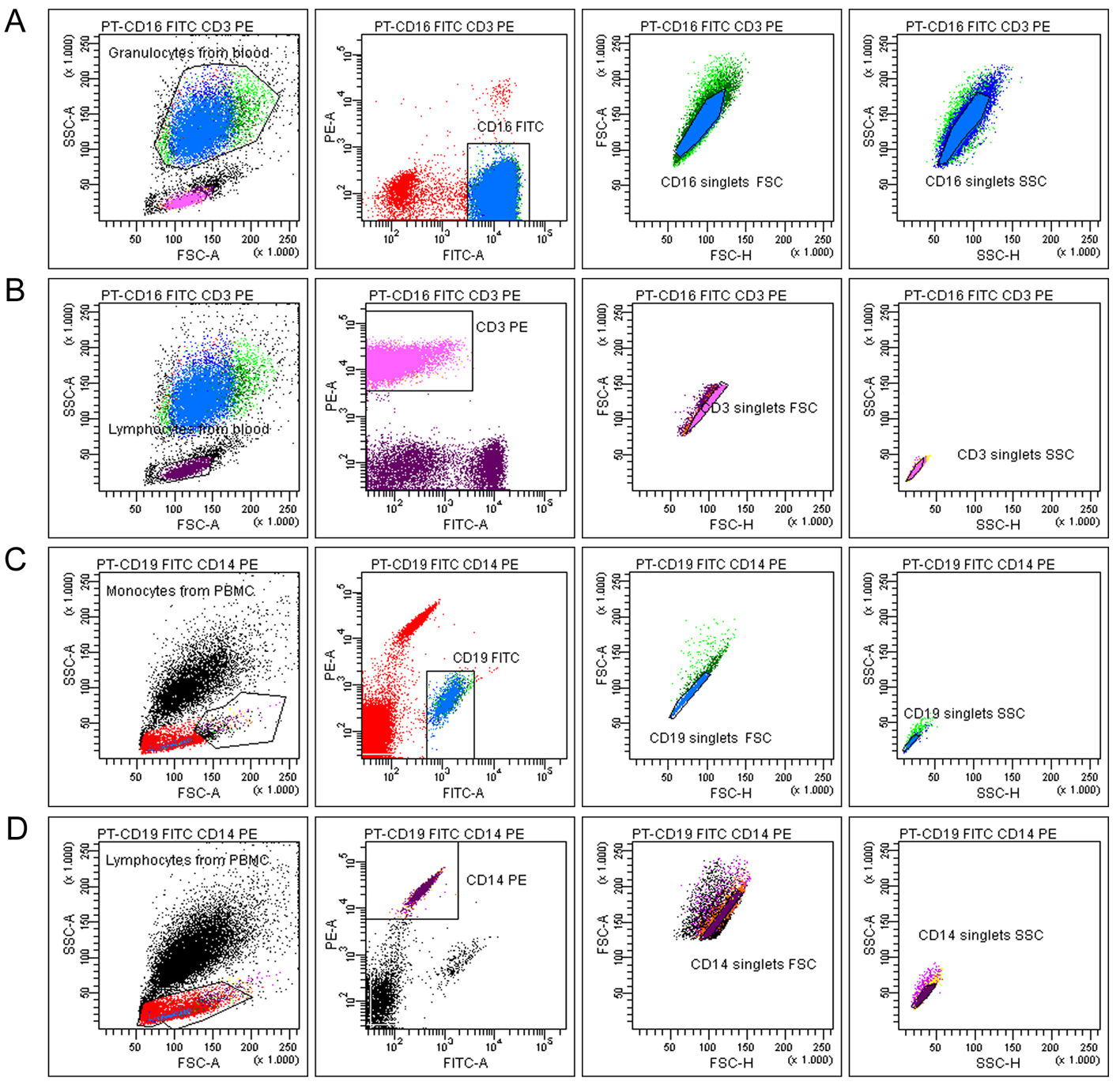

Supplement: Figure S1 — Scatter plots of gating strategy for cell sorting. Scatter plots for gating strategy employed for isolation of A: CD16-FITC, B: CD3-PE, C: CD19-FITC, and D: CD14-PE performed as double labelled sorting with gates for the isolation of CD16+ granulocytes and CD3+ T-lymphocytes from peripheral blood (blood) and in CD14+ monocytes and CD19+ B-lymphocytes isolated from peripheral blood mononuclear cells (PBMC). Back gating for separation of labelled cells and single cell isolation in forward- and side scatter plots is illustrated with colours (CD16+/CD3+ singlets: blue/pink, CD19+/CD14+ singlets: blue/pink. Cells were gated for cell type on FSC/SSC plot prior to flourophore gating. Singlets were obtained by gating in FSC-H/FSC-A and subsequently SSC-H/SSC-A. PBMC, peripheral blood mononuclear cells. (TIF) [file pone.0033100.s001.tif]
