# Supplementary material for: A Highly Sensitive Quantitative Real-Time PCR Assay for Determination of Mutant JAK2 Exon 12 Allele Burden
Source: PLoS One. 2012 Mar 5;7(3):e33100. doi: 10.1371/journal.pone.0033100 (PMC3293922; doi:10.1371/journal.pone.0033100)
Supplement: Table S1 — Clinical features of PV patients with JAK2 exon 12 mutations. Clinical data at time of diagnosis as indicated. Cell counts are in ×109/L. F, female; M, Male; Diagn., diagnosis; PV, polycythemia vera; Years, years from diagnosis; %mut, JAK2 exon 12 mutant allele burden at time of cell sorting; Hct, hematocrit; Hb, haemoglobin; Wbc, white blood cell count; Trc, platelet count; Epo, erythropoietin; Norm., normal; Spleen, spenomegaly; Thromb, previous thromboses; BM, bone marrow; EEC, Epo-independent Endogenous Colony -growth; Seq, sequencing; Treat, treatment; Hu, hydroxyurea; V, venesection; A, Aspirin. * PV3 and PV6 later developed splenomegaly and PV6 had an incidence of transient cerebral ischemic attack. (PPT) [file pone.0033100.s002.ppt]

## Slide 1
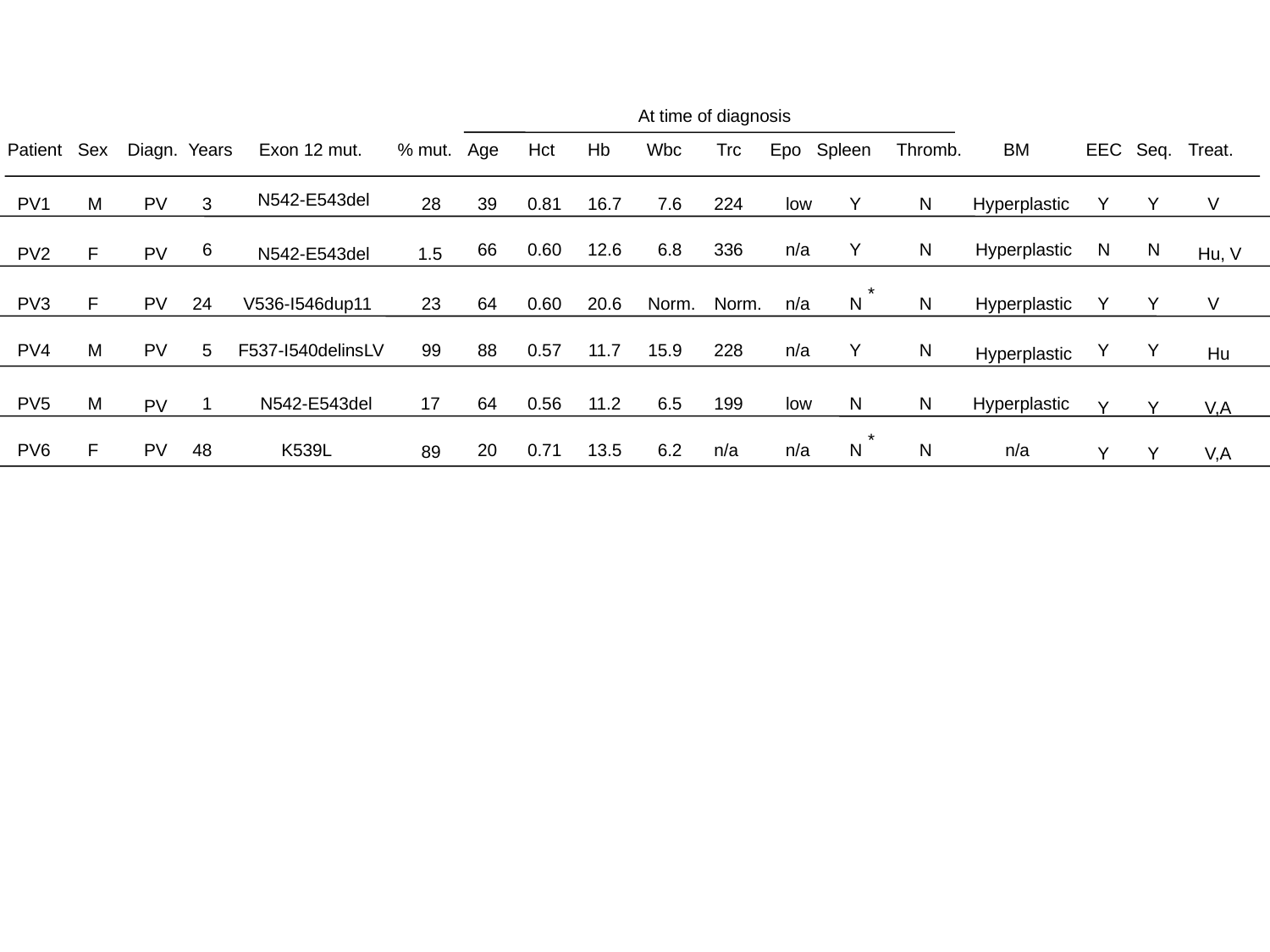

At time of diagnosis
Patient
Sex
Diagn.
Years
Exon 12 mut.
% mut.
Age
Hct
Hb
Wbc
Trc
Epo
Spleen
Thromb.
BM
EEC
Seq.
Treat.
N542-E543del
3
39
0.81
16.7
7.6
224
low
Y
N
Hyperplastic
PV1
M
PV
| 28 |
| --- |
Y
Y
V
6
66
0.60
12.6
6.8
336
n/a
Y
N
Hyperplastic
N
N
PV2
F
PV
N542-E543del
| 1.5 |
| --- |
Hu, V
*
PV3
F
PV
24
V536-I546dup11
| 23 |
| --- |
64
0.60
20.6
Norm.
Norm.
n/a
N
N
Hyperplastic
Y
Y
V
PV4
M
PV
5
F537-I540delinsLV
88
0.57
11.7
15.9
228
n/a
Y
N
Y
Y
| 99 |
| --- |
Hyperplastic
Hu
PV5
M
1
N542-E543del
| 17 |
| --- |
64
0.56
11.2
6.5
199
low
N
N
Hyperplastic
PV
Y
Y
V,A
*
PV6
F
PV
48
K539L
20
0.71
13.5
6.2
n/a
n/a
N
N
n/a
| 89 |
| --- |
Y
Y
V,A
